# Death of Dr. George McClellan

**Published:** 1847-06

**Authors:** 


### DEATH OF DR. GEORGE M'CLELLAN.

"This community," says the editor of the Phila. American, "was yesterday plunged in the most profound grief by intelligence of the death of one of its most distinguished and estimable citizens, Dr. G. McClellan. This deplorable event took place very suddenly on Saturday night. On the morning of that day he called at our office, apparently in the enjoyment of his usual health, and manifested all the vivacity and activity which characterised him; but in the afternoon we learn that he was taken suddenly ill with a bilious colic, which terminated his life in the course of the night. The news fell with startling suddenness upon the community in which he had been so long and so universally known and cherished. Hundreds of families were wrapped in the deepest affliction, and all felt and lamented the blow as one which deprived the city of one of its greatest benefactors and the country of one of its brightest ornaments. Our community did not comprise an individual more respected or beloved, one whose genius was more admired, or whose estimable personal qualities were more beloved; and the loss which has been sustained will be lamented by almost every heart and around every hearthstone in the city."

Dr. McClellan was a native of New England, a graduate of the University of Pennsylvania, and one of the founders of the Jefferson Medical College. As an operative surgeon he enjoyed a high reputation, and he was engaged in preparing a textbook on surgery, the first volume of which was to have been published the day on which his death was announced. He died in the fifty-first year of his age, in the midst of his usefulness.

